# First Record of Flower Bud Galls in *Senega* (Fabales: Polygalaceae): The Case of *S. salasiana* and Their Effect on Plant Reproduction

**DOI:** 10.3390/plants14091337

**Published:** 2025-04-29

**Authors:** Agustina Martinez, Nicolás Kuzmanich, Alejandro Farji-Brener

**Affiliations:** 1 Instituto de Investigaciones en Biodiversidad y Medioambiente (INIBIOMA), Universidad Nacional del Comahue-CRUB, CONICET, Bariloche 8400, Argentina; 2 Instituto Multidisciplinario de Biología Vegetal-CONICET, Edificio de Investigaciones Biológicas y Tecnológicas, Córdoba 5000, Argentina; nicolaskuzmanich@gmail.com; 3 Instituto de Investigaciones en Biodiversidad y Medioambiente (INIBIOMA, LIHO), Universidad Nacional del Comahue-CRUB, CONICET, Bariloche 8400, Argentina; alefarji@yahoo.com

**Keywords:** Acari, Cecidomyiidae, Diptera, florivory, herbivory, Patagonia, *Polygala*

## Abstract

We report the first case of flower bud galls in a species of the mainly American genus *Senega* (Polygalaceae), specifically in the South Andean Patagonian plant species *S. salasiana*. The galls were found to be induced by gall midges (Diptera, Cecidomyiidae) and mites (Acari). We also assessed their impact on plant reproduction by measuring the relationship between inflorescence density (number of inflorescences/plant cover) and gall abundance in two populations next to San Carlos de Bariloche, Patagonia, Argentina. Plant reproduction was negatively related to the number of galled inflorescences, suggesting that high levels of gall abundance strongly reduced plant reproductive success. Our study illustrates a novel case of flower galls in the genus *Senega* and highlights the negative impact of this type of herbivory on plant fitness.

## 1. Introduction

Plants face different kinds of herbivory that may differentially affect their fitness. One well-documented example is foliar herbivory, where leaf loss can significantly reduce a plant’s ability to photosynthesize and allocate resources to reproduction [1]. This can lead to decreased flower production, lower seed set, and reduced overall reproductive success [2]. Unlike leaf-feeding herbivores, gall-inducing organisms modify plant tissues by creating novel structures called galls, which can alter resource allocation, disrupting normal development and impacting reproductive structures. While some studies have explored these effects [3,4,5], more research is needed to fully understand how galls influence plant fitness.

Galls are a type of herbivory in which an organism induces a tissue reorganization in a plant organ and gives place to a new structure that provides food, shelter, and/or protection to the gall inducer and its progeny [6]. Galls can take place in different organs of the plants, affecting their growth and reproduction [7]. They are usually located in leaves, stems, roots, and flowers [8,9,10]. A less common type of gall affects the whole inflorescence, as is the case reported here for the first time in a species of the mainly American genus *Senega*.

*Senega* species (Polygalaceae) are known for their medicinal properties [11]. However, they have only gained importance in recent years, with the systematic and phylogenetic studies led mostly by Pastore [12,13,14], which resulted in the segregation of the genus from the traditional *Polygala* Old World Clade [15]. This work was carried out in collaboration with Martinez et al. [16,17], who included the Andean Patagonian species.

This recently addressed gap in basic taxonomic information on the Andean Patagonian *Senega* species explains the lack of studies related to their biological interactions, ecology, and phytochemistry, which are critical for understanding the group. Currently, herbivory studies in the whole plant family are scarce, with only a few documented cases of galls. In the Americas, there are three reported cases of Polygalaceae species hosting gall-inducing organisms. Stem galls were caused by an unidentified gall inducer in the Brazilian shrub *Bredemeyera floribunda* [18]; flower bud galls were induced by Cecidomyiidae (Diptera) in the genus *Securidaca* [19], and stem galls were caused by Lepidoptera (*Saphenista muerta*) in Costa Rica’s shrub *Monnina crepinii* [20]. In Argentina, where eight genera and 61 Polygalaceae species occur [17], no records of this interaction exist in the literature. In South Africa, the native *Polygala myrtifolia* and related Polygalaceae species have been extensively studied to identify phytophagous organisms for potential biological control of this plant in Australia. These studies reported bud galls in the leaves and flowers of *P. myrtifolia*, *P. virgata*, and *P. leptophylla*; as well as stem galls in *Muraltia spinosa* and *M. heisteria* and the Australian species *P. japonica* and *Comesperma volubile*, all of them caused by Acari of the genus *Aceria* (*A. myrtifoliae* and *A. virgatae*) [21,22]. The same study also reported that Cecidomyiidae (Diptera) of the genus *Dasineura* induce galls in the South African *P. peduncularis* and *P. teretifolia*, leading to stunted stem growth and, in some cases, death of the stem meristems [21].

*Senega salasiana* is a prostrate herbaceous species endemic to Argentina and Chile, distributed along the Andes from approximately 30° S Coquimbo (Chile) to the southernmost regions of South America in Tierra del Fuego (Argentina). It occupies a wide elevational range, growing from sea level up to 2900 m a.s.l. on slopes and mountain peaks. This species is characterized by its small (4 to 5 mm), zygomorphic flowers arranged in “capituliform” racemes, typically comprising around seven flowers per inflorescence. The spathulate, glabrous leaves may sometimes extend beyond the inflorescence (for further details and photographs, see Martinez [17]). In San Carlos de Bariloche, *S. salasiana* populations occur very isolated and are scarce (A. Martinez, personal observation). In this region, populations remain under snow during the winter months and begin to emerge in late October. Flowering starts in mid-November, with the earliest blooms observed in the eastern populations. By mid-December, fruit development is underway, and by early February, most of the capsules with their seeds fall.

Despite the limited research on herbivory in Polygalaceae, no studies have examined the effects of the galling organism on plant reproduction. Galls are frequently linked to decreased plant growth and reproduction. It has been suggested that galled plants produce fewer flowers and fruits, resulting in lower reproductive success [23,24]. Furthermore, the negative impacts of galling organisms can be more pronounced when gall induction occurs during the early stages of the reproductive season. Additionally, galls near the reproductive organs can significantly affect fruit and seed production, directly influencing the host plant’s fitness [7].

Here, we studied the interactions between two galling organisms and *S. salasiana* (Polygalaceae) with the aim of describing the gall morphotypes and assessing the effect of gall abundance on plant reproduction in two populations near San Carlos de Bariloche, Rio Negro, and Patagonia, Argentina. If galls negatively affect plant reproduction, we expect that inflorescence density decreases as the gall abundance per plant increases.

## 2. Results

### 2.1. Galls Description

**Figure 1:**
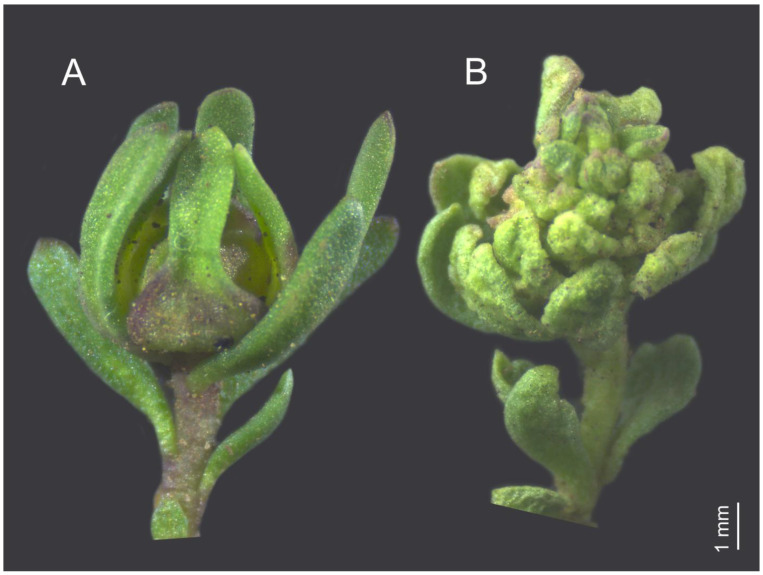
Gall morphotypes. (**A**) Diptera bud gall, a rosette-like structure of overlapping abnormal leaves, which are thickened and bunched together to form a bulbous swelling around 5 mm in diameter. (**B**) Acari bud gall, an irregular and amorphous structure, stunted, fleshy, crinkly terminal, and irregularly thickened leaves forming an amorphous rosebud-like terminal structure.

**Figure 2:**
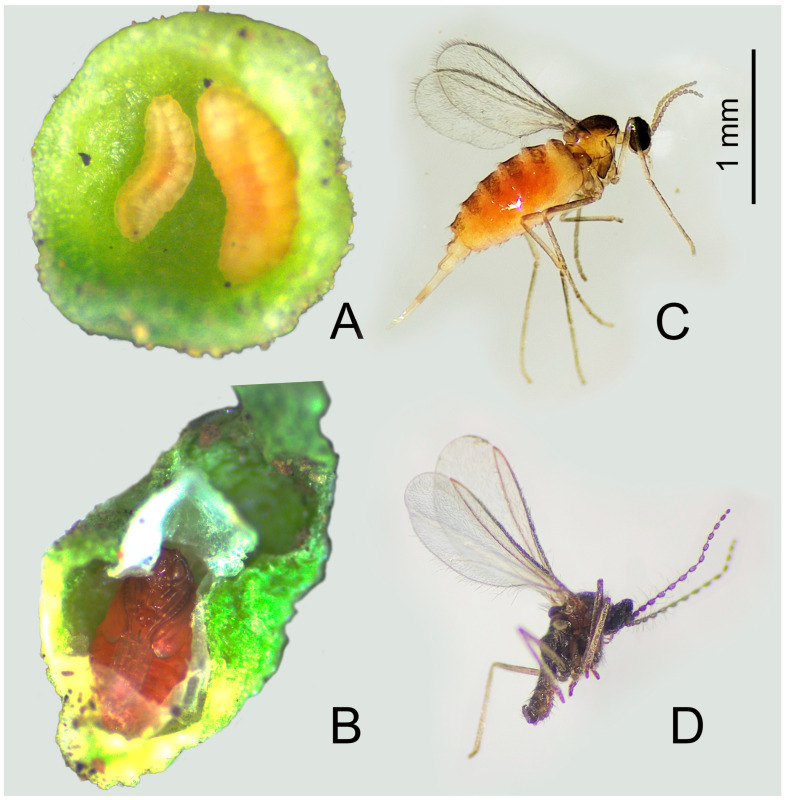
Cecidomyiidae (Diptera) life stages observed. (**A**) Two larvae on a hypertrophied leaf. (**B**) Pupae with part of the cocoon removed. (**C**) Adult female. (**D**) Adult male.

In *S. salasiana*, we identified two distinct flower gall morphotypes. The first morphotype (Diptera) was found in both populations (Cerro Villegas and Cerro San Martin), while the second morphotype (Acari) was observed exclusively in the Cerro San Martin population. Single plants hosting mixed galls induced by both agents were frequently observed. Galls were found in the early stages of plant development, forming in flower buds, and continued to be present throughout the plant’s reproductive season, eventually drying up and falling off. The first morphotype is a well-defined, rosette-like structure of overlapping abnormal leaves that are thickened and bunched together to form a bulbous swelling of around 5 mm in diameter (Figure 1A). This gall morphotype was found to be induced by Cecidomyiidae (Diptera). Each gall contained two to five larvae, each one residing in a cup-like cavity at the center of the abnormal leaves surrounding the shoot meristem. The larvae later pupated inside silky cocoons on these abnormal leaves (Figure 2).

**Figure 3:**
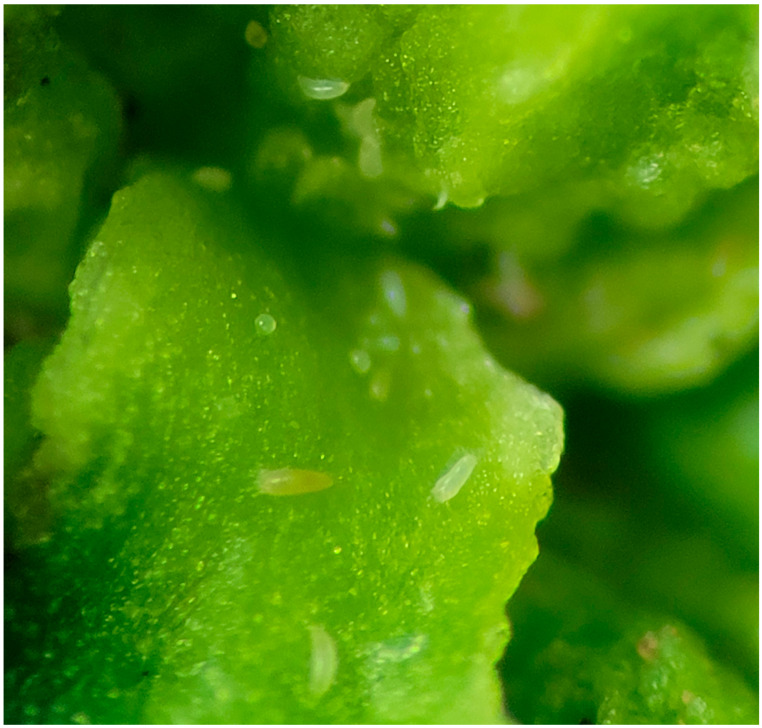
White mites (Acari) and their eggs were observed on bud galls.

The second morphotype is an irregular and amorphous growth of the flower bud. It is characterized by stunted, fleshy, crinkled terminal and irregularly thickened leaves forming a distorted rosebud-like terminal structure (Figure 1B). This gall type was induced by whitish mites (Acari: Eriophyinae: Acerini), which were observed under a magnified glass within the hypertrophied inflorescences (Figure 3). 

**Figure 4:**
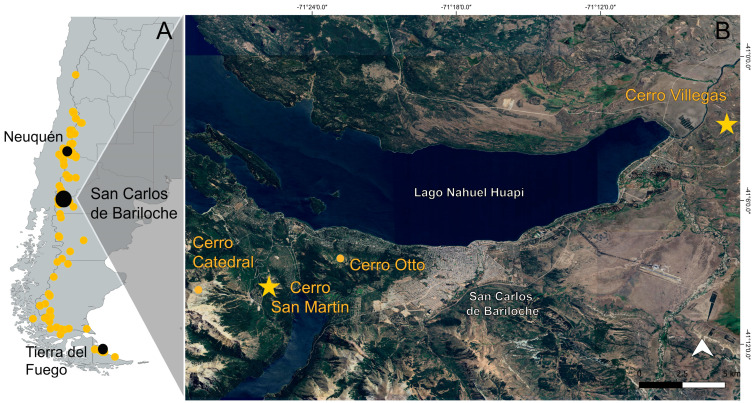
(**A**) Known distribution of *Senega salasiana* in Patagonia indicated by yellow dots (adapted from [17]). Black dots indicate gall occurrences in San Carlos de Bariloche, documented in this study, along with additional records from iNaturalist in Neuquén and Tierra del Fuego provinces [26,27]. (**B**) Populations of *S. salasiana* analyzed in this study around San Carlos de Bariloche. Yellow stars indicate the populations studied; yellow dots represent populations where no galls were found.

Through the citizen science platform iNaturalist [25], we identified at least one record of galls in *S. salasiana* from Tierra del Fuego province, Argentina [26], and another from the department of Minas, Neuquén province, Argentina [27] (Figure 4).

### 2.2. Effect on the Number of Inflorescences

The mean gall abundance per plant was 5 ± 0.43 (Mean ± SE). Most plants hosted three galls (16%), while a small proportion (2%) had a maximum of 22 galls.

**Figure 5:**
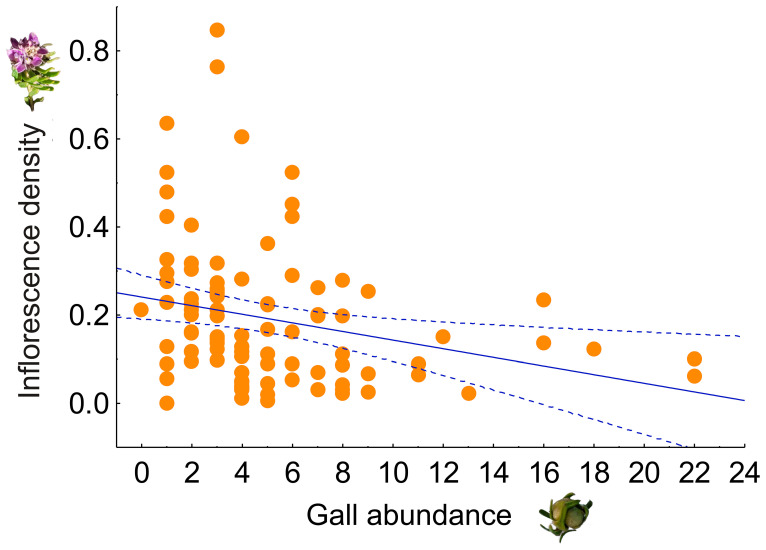
Relationship between the gall abundance per plant and the inflorescence density per plant in *Senega salasiana* in both populations (Villegas and San Martin) (F_1,96_ = 6.6, *p* = 0.01). Each dot represents a single plant observation (n = 96). Solid blue line indicates a negative linear regression between gall abundance and inflorescence density across all observations. Dashed blue lines indicate the confidence intervals (96%) around the regression line.

The inflorescence density was negatively affected by the gall abundance (F_1,96_ = 6.6, *p* = 0.01). Plants with fewer than six galls showed a high variation in the inflorescence density, whereas those with more than eight galls consistently showed lower inflorescence density (Figure 5).

## 3. Discussion

This study presents two key findings. First, it provides the first quantitative data on previously unknown interactions between a plant and two galling organisms in Patagonia. Second, it confirms that galls have a negative effect on plant reproduction, especially in plants with high gall abundance.

The discovery of this novel interaction highlights the importance of studying plant–animal interactions, as previously unknown species or host relationships may exist, especially in underexplored places such as Patagonia. Given that new species and even genera continue to be identified within Cecidomyiidae [28,29,30,31,32] and Acari [33,34,35,36], further research is necessary to clarify the taxonomic identity of the gall-inducing species on *S. salasiana*. Identifying these organisms would enhance our understanding of their evolutionary history, biogeographical distribution, life cycles, and ecological interactions with their host plant and the broader ecological community.

The galls observed on *S. salasiana* in the Argentinian Patagonia closely resemble those reported by Adair et al. [21] for the South African Polygalaceae species. Specifically, the gall morphotype induced by Cecidomyiidae (Diptera) shares striking similarities with those caused by *Dasineura* sp., while the second morphotype induced by Acari is comparable to the galls caused by *Aceria* sp. [21]. This morphological resemblance suggests possible convergent evolution or a historical biogeographical connection between these gall-inducing taxa across continents.

Through records obtained via the citizen science platform iNaturalist [25], we documented at least one observation of galls on *S. salasiana* in Tierra del Fuego, the southernmost distribution of the species [26]. Additionally, another observation was recorded in the Minas department, Neuquén province, Argentina [27], at almost the northern limit of its range (Figure 4). These records confirm the presence of the gall–plant interaction across nearly the entire distribution of *S. salasiana*. This broad geographic occurrence raises questions about potential evolutionary relationships between the plant and its gall-inducing organisms, warranting further investigation, such as whether the same galling species are present throughout the entire plant’s range or if different species are present in different regions. Additionally, the role of environmental factors like climate and elevation in shaping the distribution and intensity of gall formation remains to be explored.

Galls can affect plant fitness in several ways, depending on their location and severity. When they develop on leaves, they can reduce the plant’s photosynthetic capacity, thereby limiting the resources available for reproductive structures such as flowers and seeds. However, in this case, the galls exclusively affect the inflorescences, having a direct impact on reproduction. By atrophying the complete inflorescence, these galls can obstruct pollination in multiple ways: physically reducing the number of flowers available for pollinators and potentially altering the visual and chemical cues that attract pollinators, leading to reduced visitation rates.

The negative impact on plant reproduction suggests that gall formation could influence *S. salasiana* population dynamics, potentially reducing seed production and altering plant fitness. This may have consequences on the local ecosystem, especially if *S. salasiana* serves as a resource for other organisms. For example, in Cerro Villegas, *S. salasiana* flowers have been observed as a food source for *Pseudomeloe* beetles, a previously unreported interaction [37]. This highlights the plant’s ecological role in supporting both herbivores and pollinators. Furthermore, as *S. salasiana* seeds appear to be adapted for ant-mediated dispersal, reduced seed availability due to galling might also impact the assembly of seed and dispersers. Further research is also needed to investigate the presence of parasitoids associated with gall-inducing arthropods. Identifying these factors could provide valuable insight into the broader ecological dynamics of gall formation and its impact on both the host plant and associated species.

## 4. Materials and Methods

### 4.1. Study Area

In San Carlos de Bariloche, Río Negro province, Argentina, *S. salasiana* populations were searched using herbarium records published in a recent taxonomic revision of *Senega* subg. *Clinclinia* [17], as well as observations from the citizen science platform iNaturalist [25]. Four populations above 1100 m a.s.l., from late October 2024 to the beginning of February 2025, were found. From East to West: 1. Cerro Villegas; 2. Cerro Otto; 3. Cerro San Martín; and 4. Cerro Catedral. Galled plants were found only in populations from Cerro Villegas and Cerro San Martin. Thus, the only populations analyzed in this study were Cerro Villegas and Cerro San Martin (Figure 4).

1. Cerro Villegas 41°02′51″ S, 71°06′44″ W, 1175 to1202 m a.s.l., measured 28 November 2024. The site vegetation showed a predominance of *Acaena* sp. and grasses, with the presence of *Astragalus* sp., *Adesmia* sp., and *Viola maculata.* The population area measured was approximately 3240 m^2^;
2. Cerro San Martin, 41°9′36″ S, 71°25′45″ W, 1260 to 1266 m a.s.l., measured 8 and 17 December 2024. The site vegetation showed a predominance of *Anemone multifida*, *Quinchamalium* sp., *Acaena* sp., *Alstroemeria aurea*, and *Berberis microphylla*. The population area measured was approximately 3080 m^2^.

### 4.2. Methodology

Through field observations, we searched for galls in *S. salasiana*. Sample galls were collected, observed, and photographed through a magnifying glass. In Acari galls, a gall sample was kept in 70% ethanol together with a herbarized gall sample. In the laboratory, Diptera galls, along with some larvae and pupae, were kept in glass jars under natural environmental conditions for approximately 1 to 7 days until adults emerged. Larvae, pupae, and adult stages were photographed (Figure 2) and kept in 70% ethanol together with a herbarized gall sample. All samples were deposited at the Museo Argentino de Ciencias Naturales “Bernardino Rivadavia” (MACN) in Buenos Aires. Vouchers were identified as “A. Martinez 1 to 3” for Diptera and “A. Martinez 4” for Acari. Galling inducers were identified at the highest level of taxonomic resolution possible by comparison with reference material and identification keys available in the literature [28,38].

### 4.3. Data Collection

#### 4.3.1. Gall Abundance per Plant, Number of Inflorescences, and Plant Cover

**Figure 6:**
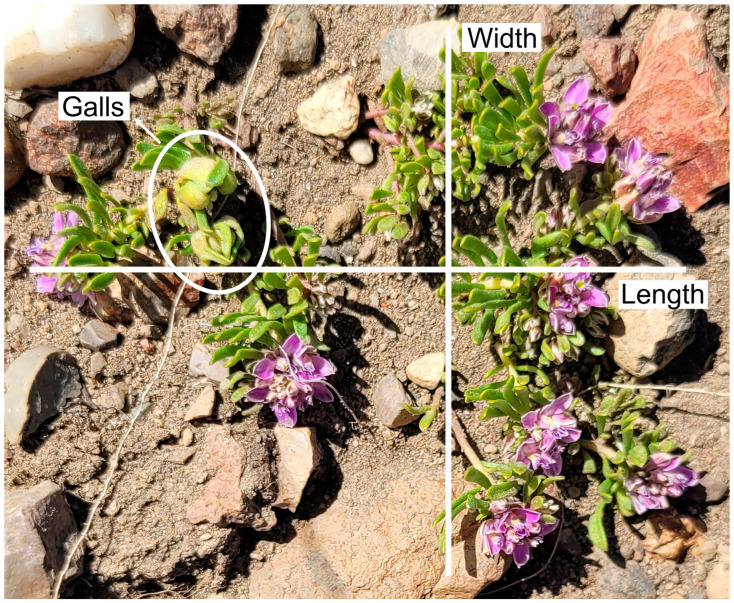
*Senega salasiana* with Cecidomiidae galls. The number of inflorescences is 8, and the number of galls is 2. Also, length and width are depicted as measured for this study.

*S. salasiana* plants were inspected for galls during the spring of 2024. We found 98 plants, and for each plant, we measured the number of galls (gall abundance), the number of inflorescences, and the plant cover. The plant cover was estimated as an ellipse, measuring the length and width of the plant with a hand ruler (Figure 6, Table S1).

#### 4.3.2. Data Analysis

To determine the effect of gall abundance on plant reproduction, we conducted a linear regression using the software Statistica^®^ Version 8.0 between the number of galls per plant as the independent variable and the inflorescence density (i.e., the number of inflorescences per plant/plant cover) as the dependent variable (Figure 5).

## Data Availability

The original contributions presented in this study are included in the article/Supplementary Material. Further inquiries can be directed to the corresponding author(s).

## Supplementary Materials

The following supporting information can be downloaded at https://www.mdpi.com/article/10.3390/plants14091337/s1, Table S1. Gall abundance and inflorescence density per plant during November and December 2024.

## References

[1] Marquis R.J. (1984). Leaf Herbivores Decrease Fitness of a Tropical Plant. Science.

[2] Viana L.R., Silveira F.A.O., Santos J.C., Rosa L.H., Cares J.E., Café-Filho A.C., Fernandes G.W. (2013). Nematode-induced galls in *Miconia albicans*: Effect of host plant density and correlations with performance. Plant Species Biol..

[3] Silva I.M., Andrade G.I., Fernandes G.W., Lemos Filho J.P. (1996). Parasitic Relationships between a Gall-forming Insect *Tomoplagia rudolphi* (Diptera: Tephritidae) and its Host Plant (*Vernonia polyanthes*, Asteraceae). Ann. Bot..

[4] Petro R., Madoffe S.S., Iddi S., Mugasha W.A. (2015). Impact of Eucalyptus gall wasp, *Leptocybe invasa* infestation on growth and biomass production of *Eucalyptus grandis* and *E. saligna* seedlings in Tanzania. Int. J. Pest Manag..

[5] Strydom M., Veldtman R., Ngwenya M.Z., Esler K.J. (2024). Questioning the effectiveness of seed-reducing agents on invasive *Acacia*: Pod production relative to gall abundance of classical biological control agents. Perspect. Plant Ecol. Evol. Syst..

[6] Shorthouse J.D., Wool D., Raman A. (2005). Gall-inducing insects—Nature’s most sophisticated herbivores. Basic Appl. Ecol..

[7] Barrancos M.L., Moncaglieri R., Farji-Brener A. (2008). Infección por agallas y producción de inflorescencias en el arbusto *Schinus patagonicus*. Ecol. Austral..

[8] Mani M.S., Weisbach W.W., Van Oye P. (1964). Ecology of Plant Galls.

[9] Kuzmanich N., Altamirano A.A., Salvo A. (2015). Agallas de insectos de la región Rioplatense, Buenos Aires. Rev. Soc. Entomol. Argent.

[10] Kuzmanich N., Giorgis M.A., Salvo A. (2018). Insect galls from Córdoba, Argentina: A case where stem galls predominate. Rev. Biol. Trop..

[11] (2025). Medicinal Plant Names Services Portal, Royal Botanic Gardens, Kew, Version 14. http://mpns.kew.org/mpns-portal.

[12] Pastore J.F.B., Abbott J.R., Neubig K.M., Van Den Berg C., Mota M.C.D.A., Cabral A., Whitten W.M. (2019). Phylogeny and biogeography of *Polygala* (Polygalaceae). Taxon.

[13] Pastore J.F.B., Mota M., Amano E., Martinez A. (2021). Disentangling *Polygala obovata* Complex (Polygalaceae), with a Description of Three New Species for Brazil. Syst. Bot..

[14] Pastore J.F.B. (2022). Revision of the ‘*Polygala herbiola* group’ (Polygalaceae): A new species and a new variety. Kew Bull..

[15] Pastore J.F.B., Martinez A., Abbott J.R., Neubig K. (2023). Toward New Generic Delimitations in Polygalaceae II: *Senega*. Ann. Mo. Bot. Gard..

[16] Martinez A., Acosta J.M., Ferrero M.A., Pastore F.B., Aagesen L. (2022). Evolutionary patterns within the New World Clade *Polygala* sections *Clinclinia* and *Monninopsis* (Polygalaceae). Perspect. Plant Ecol. Evol. Syst..

[17] Martinez A. (2023). Revisión de *Senega* subgénero *Clinclinia* (Polygalaceae). Darwiniana Nueva Ser..

[18] Urso-Guimarães M.V., Scareli-Santos C. (2006). Galls and gall makers in plants from the Pé-de-Gigante Cerrado Reserve, Santa Rita do Passa Quatro, SP, Brazil. Braz. J. Biol..

[19] Ramos Rodrigues A., Maia V.C., Souto Couri M. (2014). Insect galls of restinga areas of Ilha da Marambaia, Rio de Janeiro, Brazil. Rev. Bras. Entomol..

[20] Nishida K., Adamski D. (2004). Two new gall-inducing *Saphenista* Walsingham (Lepidoptera: Tortricidae: Cochylini) from Costa Rica. Proc. Entomol. Soc. Wash..

[21] Adair R.J., Neser S., Stajsic V. (2011). Phytophagous Organisms Associated with the Woody Shrub “*Polygala myrtifolia*” (Polygalaceae) and Their Potential for Classical Biological Control in Australia. Plant Prot. Q..

[22] Smith Meyer M.K.P., Ueckermann E.A. (1996). Three new species of *Aceria* (Acari: Eriophyidae) from South African Polygalaceae and Polygonaceae. Int. J. Acarol..

[23] Martini V.C., Raymundo D., Prado-Junior J., Oliveira D.C. (2021). Bottom-up and top-down forces in plant-gall relationships: Testing the hypotheses of resource concentration, associational resistance, and host fitness reduction. Ecol. Entomol..

[24] Leege L.M. (2006). The relationship between psyllid leaf galls and redbay (*Persea borbonia*) fitness traits in sun and shade. Plant Ecol..

[25] iNaturalist (2025). https://www.inaturalist.org/home.

[26] Castillo C. (2021). Observation of *Senega salasiana*. In iNaturalist. https://www.inaturalist.org/observations/102924729.

[27] Sersic A. (2007). Observation of *Senega salasiana*. In iNaturalist. https://www.inaturalist.org/observations/103823852.

[28] Gagné R.J., Jaschof M. (2021). A Catalog of the Cecidomyiidae (Diptera) of the World.

[29] Kolesik P., McFadyen R.E.C., Wapshere A.J. (2000). New gall midges (Diptera: Cecidomyiidae) infesting native and introduced *Solanum* spp. (Solanaceae) in Australia. Trans. R Soc. S Aust. Inc..

[30] Kolesik P., Sutton G.F., Steenderen C.J.M., Martins D.J., Plowes R., Paterson I.D. (2025). A new genus and two new species of gall midges (Diptera: Cecidomyiidae) feeding on Guinea grass *Megathyrsus maximus* (Poaceae) in Africa. Austral. Entomol..

[31] Kolesik P., Sutherland R., Gillard K., Gresham B., Withers T.M. (2021). A new species of *Mycodiplosis* gall midge (Diptera: Cecidomyiidae) feeding on myrtle rust *Austropuccinia psidii*. N. Z. Entomol..

[32] Kolesik P., Adair R.J., Eick G. (2005). Nine new species of *Dasineura* (Diptera: Cecidomyiidae) from flowers of Australian *Acacia* (Mimosaceae). Syst. Entomol..

[33] Flechtmann C.H.W., De Queiroz D.L. (2010). New taxa in the Eriophyidae (Acari, Prostigmata) from forest trees in southern Brazil. Zootaxa.

[34] Chetverikov P.E., Desnitskiy A.G., Letukhova V.Y., Ozman-sullivan S.K., Romanovich A.E., Sarratt J.V., Sukhareva S.I. (2021). A new species, new records, and DNA barcodes of eriophyine mites (Eriophyidae, Eriophyinae) from southeast Crimea and remarks on ability to form galls in conspecific eriophyoids. Syst. Appl. Acarol..

[35] Khaustov A.A., Fjellberg A., Lindquist E.E. (2022). A new genus and species of Pseudotarsonemoidini (Acari: Heterostigmata: Tarsonemidae) associated with xylophagous gall midges in Norway. Syst. Appl. Acarol..

[36] Situngu S., Elhalawany A.S., Ngubane-Ndhlovu N.P., Chetverikov P.E. (2023). New species and records of gall mites of the genus *Aceria* (Eriophyoidea, Eriophyidae) associated with *Tamarix* in Egypt and South Africa. Acarologia.

[37] Safenraiter M.E., Campos-Soldini M.P., Fernández E.E.N., Del Rio M.G. (2019). Escarabajos vesicantes Sudamericanos (Coleoptera: Meloidae). Aportes al estado del conocimiento del género andino *Pseudomeloe* Fairmaire y Germain. Idesia Arica.

[38] Manson D.C.M. (1984). Eriophyinae (Arachnida: Acari: Eriophyoidea). Fauna N. Z..

