# Supplementary material for: First Record of Flower Bud Galls in Senega (Fabales: Polygalaceae): The Case of S. salasiana and Their Effect on Plant Reproduction
Source: Plants (Basel). 2025 Apr 29;14(9):1337. doi: 10.3390/plants14091337 (PMC12073542; doi:10.3390/plants14091337)
Supplement: Supplementary file 1 [file plants-14-01337-s001.zip › plants-3582711-supplementary.pdf]

Supplementary Materials

Table S1. Gall abundance and inflorescence density per plant during November and December 2024

Locality - date	Plant	Length (cm)	Width (cm)	n. inflore scences	Plant cover	Gall abundance	Inflorescence density
Villegas - 28 Nov.	V1	30	12	56	282.7433388	7	0.198059485
	V2	6	7	8	32.98672286	3	0.242521818
	V3	19	15	26	223.8384766	4	0.116155187
	V4	25	17	8	333.7942194	13	0.023966862
	V5	24	13	8	245.044227	7	0.032647168
	V6	30	19	123	447.6769531	3	0.274751691
	V7	22	8	44	138.2300768	2	0.318309886
	V8	20	13	57	204.2035225	8	0.279133285
	V9	14	13	34	142.9424657	2	0.237857937
	V10	13	7	12	71.47123287	5	0.16789972
	V11	24	7	17	131.9468915	1	0.128839716
	V12	30	6	8	141.3716694	1	0.056588424
	V13	30	8	85	188.4955592	6	0.450939005
	V14	30	14	66	329.8672286	7	0.2000805
	V15	6	13	37	61.26105675	4	0.603972605
	V16	7	5	9	27.48893572	1	0.327404454
	V17	30	6	37	141.3716694	7	0.261721462
	V18	7	4	14	21.99114858	1	0.636619772
	V19	19	7	17	104.4579557	6	0.162744904
	V20	10	11	24	86.39379797	1	0.277797719
	V21	18	18	13	254.4690049	4	0.051086772
	V22	25	11	8	215.9844949	4	0.037039696
	V23	14	3	5	32.98672286	3	0.151576136
	V24	40	12	38	376.9911184	22	0.100798131
	V25	25	15	36	294.5243113	3	0.122230996
	V26	25	15	18	294.5243113	22	0.061115498
	V27	11	6	21	51.83627878	2	0.405121673
	V28	29	10	45	227.7654674	3	0.197571653
	V29	14	13	16	142.9424657	8	0.111933147
	V30	25	15	75	294.5243113	9	0.254647909
	V31	7	6	3	32.98672286	1	0.090945682
	V32	14	13	32	142.9424657	5	0.223866294
	V33	38	26	119	775.9733854	4	0.153355775
	V34	36	28	93	791.6813487	2	0.117471506
	V35	27	11	58	233.2632545	3	0.248646106
	V36	28	6	30	131.9468915	2	0.227364204
	V37	10	6	14	47.1238898	1	0.297089227
	V38	10	9	37	70.68583471	6	0.523442924
San Martin - 8 Dec.	SMA1	14	11	12	120.9513172	3	0.099213471
	SMA2	18	13	13	183.7831702	7	0.07073553
	SMA3	7	13	5	71.47123287	7	0.069958217
	SMA4	38	10	2	298.4513021	5	0.006701261
	SMA5	14	4	23	43.98229715	1	0.52293767
	SMA6	8	7	14	43.98229715	3	0.318309886
	SMA7	10	5	30	39.26990817	3	0.763943727
	SMA8	10	6	40	47.1238898	3	0.848826363
	SMA9	30	18	37	424.1150082	8	0.087240487
	SMA10	10	18	5	141.3716694	4	0.035367765
	SMA11	11	6	22	51.83627878	6	0.424413182
	SMA12	14	7	20	76.96902001	3	0.259844805

	SMA13	18	8	17	113.0973355	12	0.150313002
	SMA14	6	7	10	32.98672286	2	0.303152273
	SMA15	19	7	4	104.4579557	4	0.038292919
	SMA16	26	16	10	326.725636	8	0.03060672
	SMA17	20	8	32	125.6637061	3	0.254647909
	SMA18	6	4	4	18.84955592	3	0.212206591
	SMA19	4	3	1	9.424777961	4	0.106103295
	SMA20	30	10	21	235.619449	6	0.089126768
	SMA21	6	4	8	18.84955592	1	0.424413182
	SMA22	19	6	18	89.53539063	2	0.201037823
	SMA23	32	19	11	477.5220833	8	0.023035584
	SMA24	11	8	20	69.11503838	6	0.289372624
	SMA25	12	8	15	75.39822369	8	0.198943679
	SMA26	30	9	4	212.0575041	5	0.018862808
	SMA27	12	12	6	113.0973355	6	0.053051648
	SMA28	15	7	8	82.46680716	3	0.097008727
	SMA29	7	4	2	21.99114858	5	0.090945682
	SMA30	9	5	17	35.34291735	1	0.481001606
	SMA31	16	6	11	75.39822369	3	0.145892031
	SMA32	17	13	12	173.5729941	4	0.069135179
	SMA33	12	6	5	56.54866776	1	0.088419413
	SMA34	9	4	6	28.27433388	0	0.212206591
	SMA35	4	3	0	9.424777961	1	0
San Martin - 17 Dec.	SMB1	3	4	2	9.424777961	2	0.212206591
	SMB2	18	5	20	70.68583471	4	0.282942121
	SMB3	8	7	7	43.98229715	2	0.159154943
	SMB4	10	11	11	86.39379797	4	0.127323954
	SMB5	10	10	1	78.53981634	4	0.012732395
	SMB6	15	4	2	47.1238898	8	0.042441318
	SMB7	7	6	3	32.98672286	1	0.090945682
	SMB8	9	5	8	35.34291735	5	0.226353697
	SMB9	7	9	8	49.48008429	2	0.161681212
	SMB10	17	10	6	133.5176878	5	0.044937866
	SMB11	13	8	4	81.68140899	4	0.048970752
	SMB12	9	6	4	42.41150082	2	0.09431404
	SMB13	13	7	8	71.47123287	5	0.111933147
	SMB14	16	15	12	188.4955592	11	0.063661977
	SMB15	10	19	10	149.225651	9	0.067012608
	SMB16	20	14	30	219.9114858	16	0.136418523
	SMB17	10	7	20	54.97787144	5	0.363782727
	SMB18	13	5	7	51.05088062	3	0.137118105
	SMB19	13	9	21	91.89158512	1	0.228530175
	SMB20	20	12	23	188.4955592	18	0.12201879
	SMB21	37	6	41	174.3583923	16	0.235147844
	SMB22	26	18	60	367.5663405	6	0.163235839
	SMB23	10	8	2	62.83185307	4	0.031830989
	SMB24	20	10	4	157.0796327	9	0.025464791
	SMB25	7	4	2	21.99114858	11	0.090945682
